# P02.85. A prospective patient-centered data collection program at an acupuncture and Oriental medicine teaching clinic

**DOI:** 10.1186/1472-6882-12-S1-P141

**Published:** 2012-06-12

**Authors:** B Marx

**Affiliations:** 1 Oregon College of Oriental Medicine, Portland, USA

## Purpose

Large-scale patient-reported outcomes research investigating the role of acupuncture and Oriental medicine (AOM) in general practice is limited, despite the growing use of AOM in the United States. This paper describes the development and refinement of a prospective, patient-centered outcomes data collection program at an Oriental medicine college and presents demographic and clinical data.

## Methods

A prospective patient-centered data collection program was implemented in 2007 using the Measure Your Medical Outcomes Profile (MYMOP) questionnaire and college-developed demographic and conditions forms. The forms were completed by patients on the first and fifth clinic visit. The program was revised after two years to streamline the data entry process and to include three Patient Reported Outcome Measurement Information System (PROMIS) questionnaires measuring pain, general health, and physical functioning.

## Results

Demographics were similar to those reported in other AOM settings. The majority of patients were Caucasian females, and expressed confidence in acupuncture treatment. The most common chief complaint was joint and muscle pain. Additionally, we found that mean scores at baseline for global physical and mental health, and physical functioning were all substantially lower than US averages. In contrast to some studies, we found that a majority of patients had previous experience with acupuncture.

## Conclusion

An ongoing, prospective data collection program can be successfully developed and implemented at an AOM college. The program will ultimately provide large-scale, patient-reported outcomes on patients seeking AOM treatment at the student clinic.

